# 3.O. Workshop: Structural stigma shapes LGBTQ+ mental health and well-being across countries

**DOI:** 10.1093/eurpub/ckac129.192

**Published:** 2022-10-25

**Authors:** Organised by: University of Exeter (UK), Chair persons: David Doyle (UK)

## Abstract

Lesbian, gay, bisexual, transgender and queer (LGBTQ+) people around the world have been shown to suffer from disparities in mental health and well-being relative to cisgender heterosexual people. Past research aiming to explain these gaps has referenced the added stress experienced by LGBTQ+ people in the form of, for example, interpersonal discrimination, rejection, and harassment (i.e., minority stress). While these phenomena exist at the interpersonal level, emerging evidence suggests that discriminatory structural-level factors, such as policies, norms and rules (i.e., structural stigma), may be just as influential in shaping LGBTQ+ mental health and well-being. Given the passage of a slate of bills limiting sexual and gender minority rights and banning open speech around these issues across many different countries, this is an extremely timely issue for sexual and gender minority health. This workshop will give examples of cutting-edge research on the ways in which structural stigma affects mental health and well-being for LGBTQ+ people. Dr. Dinah Gutermuth (University of Exeter, UK) will discuss findings from a global study (conducted in partnership with the BBC) including older and younger LGB individuals from 113 different countries, showing that structural stigma impairs social capital and creates disparities in loneliness between younger and older LGB individuals. Berk Can Ünsal (Eötvös Loránd University, Hungary) will present data from Europe highlighting structural stigma as a risk factor, and community participation as a protective factor, for depression in sexual and gender minority people. Dr. John Pachankis (Yale University, USA) will present results on how migration from countries of varying levels of structural stigma shapes depression and suicidality in sexual minority men. Finally, Dr. Richard Bränström (Karolinska Instituet, Sweden), will discuss how average life satisfaction improved from 2012-2019 for sexual minority people across Europe, with greater increases in countries with higher levels of structural stigma. Together, these presentations will demonstrate the far-reaching effects of structural stigma on LGBTQ+ mental health and well-being around the world.

**Key messages:**

• Lesbian, gay, bisexual, transgender and queer (LGBTQ+) people around the world have been shown to suffer from disparities in mental health and well-being relative to cisgender heterosexual people.

• Structural stigma is an important factor determining risk for poor mental health among LGBTQ+ individuals across countries.

